# Insight to the Epidemiology and Risk Factors of Extrapulmonary Tuberculosis in Tianjin, China during 2006-2011

**DOI:** 10.1371/journal.pone.0112213

**Published:** 2014-12-10

**Authors:** Xiaoqing Wang, Zhenhua Yang, Yanyong Fu, Guoqin Zhang, Xu Wang, Yuhua Zhang, Xiexiu Wang

**Affiliations:** 1 Department of Epidemiology, School of Public Health, University of Michigan, Ann Arbor, Michigan, United States; 2 Tuberculosis Institute, Tianjin Centers for Disease Control and Prevention, Tianjin, China; Fundació Institut d'Investigació en Ciències de la Salut Germans Trias i Pujol. Universitat Autònoma de Barcelona. CIBERES, Spain

## Abstract

**Background:**

The proportion of extrapulmonary tuberculosis (EPTB) among all the reported tuberculosis (TB) cases has increased in different populations. Despite the large burden of TB in China, the epidemiology of EPTB in China remains largely understudied and the risk factors for having EPTB diagnosis in China have not been identified.

**Methods:**

To gain insight to EPTB epidemiology in China, we analyzed TB surveillance data collected in Tianjin, China, during 2006 to 2011. The frequency of EPTB among all TB cases and within different socio-demographic groups of the study patients aged 15 years and older was determined for EPTB in general and by specific types. The distribution of socio-demographic characteristics was compared between pulmonary TB (PTB) group and EPTB group by chi-square test. Crude and multiple logistic regression-derived adjusted odds ratios (aOR) and 95% confidence intervals (CI) were determined to assess the associations between having EPTB diagnosis and each individual explanatory variable in question.

**Results:**

About one-tenth (1,512/14,561) of the patients investigated in this study had EPTB. Of these 1,512 EPTB cases, about two thirds were pleural TB. Significant difference in age, occupation, and urbanity of residence were found between PTB and EPTB groups (p<0.05). Patients with EPTB diagnosis were more likely to be 65 years or older (aOR = 1.22, 95% CI: 1.02, 1.46), to be retired (aOR = 1.37, 95% CI: 1.08, 1.75), and to live in urban areas (aOR = 1 38, 95% CI: 1.22, 1.55).

**Conclusions:**

The findings of this study extends the knowledgebase of EPTB epidemiology in developing countries and highlight the need for improved EPTB detection in China, especially in subpopulations with high risk for EPTB or having limited access to medical facilities with adequate capacity for EPTB diagnosis.

## Introduction

Tuberculosis (TB) remains a global pandemic, resulting in 8.6 million new cases and 1.3 million deaths in 2012 worldwide [1]. TB ranks as the eighth leading cause of death in low- and middle-income countries [1]. More than 80% of the global burden of TB falls on 22 high-burden countries [2].

TB can occur at different anatomic sites of the human body. TB affecting the lung parenchyma is designated pulmonary TB (PTB) and TB affecting any anatomical site outside of the lung parenchyma is designated extrapulmonary TB (EPTB); the most commonly reported site is pleura [3]. Pleural TB is a form of TB within the thoracic cavity that represents relatively less dissemination of *Mycobacterium tuberculosis* infection, as compared with other forms of EPTB that are outside of thoracic cavity. PTB patients who are sputum smear positive for acid-fast bacilli are most contagious. Unlike PTB, EPTB is usually not infectious unless the patient also concurrently has PTB [3]. However, EPTB can cause death if undiagnosed or untreated, especially in immunosuppressed individuals [4].

With the rise of Human Immunodeficiency Virus (HIV) epidemic in many regions of the world, EPTB has experienced an increase in different populations as well, especially in those areas where HIV infection is increasing [5]–[10]. In the U.S. the proportion of EPTB among all the reported TB cases has increased from 15.7% in 1993 to 21.0% in 2006 [9]. However, EPTB is largely undiagnosed in developing countries, due to the difficulties in its diagnosis [3], [11]. As a result, most previous epidemiological studies of EPTB have, so far, been conducted in developed countries; there is very little information about EPTB epidemiology in developing countries, especially in those high TB-burden countries.

Among these high-burden countries, China ranks the second with an estimated incidence of 73 cases per 100,000 people in 2012 [1]. Although some epidemiologic studies have examined the socio-demographic characteristics of TB in China [12]–[16], the epidemiology and risk factors for having EPTB in China remain largely unknown. In order to extend the knowledge base of EPTB, we conducted the present study using TB surveillance data collected by Tianjin Centers for Disease Control and Prevention (TJCDC), China, between January 1, 2006 and December 31, 2011.

## Methods

### Study setting

This study was conducted in Tianjin, China. Tianjin is the fourth largest city in China with a total population of approximately 13 million, which include 11 million registered permanent residents and 2 million unregistered migrant workers [17]. It is one of the four municipalities of the People's Republic of China (PRC) that are under the direct administration of the central government of the PRC. Tianjin is divided into 18 districts. Established in 1953, TJCDC is a municipal public health institution that oversees disease prevention and control in all of these districts.

### Study sample

The study sample included a total of 14,561 (95%) of the 15,248 TB cases reported to the TJCDC's TB surveillance system during 2006 to 2011. The 687 (5%) of the reported cases that were excluded from the study sample included 567 (4%) cases for which the information on TB disease site was unavailable and 120 (1%) pediatric cases (<15 years of age). The pediatric cases were excluded from the study sample due to the consideration of a high probability for underreporting among this group that could ultimately compromise the study sample's representativeness of the pediatric patient population.

### Diagnosis of TB

The diagnosis of TB in Tianjin was done following China National TB Diagnostic Guidelines [18], which is consistent with the World Health Organization's diagnostic criteria [19]. All the cases included in this study were confirmed by laboratory-based diagnostic tests, including acid-fast- bacilli smear of specimens, culture, tuberculin purified protein derivative skin test, and serological test for *Mycobacterium tuberculosis* infection.

### Data collection

This study used a de-identified dataset derived from the TJCDC's standard electronic TB surveillance database. This dataset contained socio-demographic and clinical information of all the TB patients reported to TJCDC by health care professionals in different clinics and hospitals (including TB clinics and hospitals) located in the 18 districts of Tianjin. This study was reviewed and approved by the Health Sciences and Behavior Science Institutional Review Board of the University of Michigan and the ethical committee of TJCDC.

### Data analysis

The frequency of EPTB was calculated for overall study sample and for each socio-demographic group of the study patients, concerning the study patients' age, sex, education level, occupation, urbanity of residence, stability of residence, and the history of previous TB diagnosis. The frequency distribution of pleural TB and other forms of EPTB affecting organs and tissues outside of thoracic cavity (e.g. joint/bone, kidney) were compared among different age, education level, and occupation groups and between sex, rural and urban areas, permanent and non-permanent resident, and having and not having previous TB diagnosis groups. To identify potential risk factors for EPTB, the study patients were classified as having PTB and EPTB. PTB was defined as TB affecting the lung parenchyma. For this study, PTB was defined as TB affecting lung parenchyma, which included all the primary PTB, hematogenously disseminated PTB, and secondary PTB; EPTB was defined as TB affecting any anatomical site outside of the lung parenchyma, which included pleural TB cases and cases having TB in organs and tissues outside of thoracic cavity. The distribution of socio-demographic characteristics was compared between PTB group and EPTB group by chi-square test. P-values less than 0.05 were considered statistically significant. Crude and multiple logistic regression-derived adjusted odds ratios (OR) and 95% confidence interval (CI) were calculated to describe the strength and significance of the associations between EPTB and the variables under study. All statistical analyses were conducted using SAS version 9.3 (SAS Institute, Cary, NC).

## Results

### Patient demographic characteristics

The study patients included 9,456 (65%) males and 5,105 (35%) females. Of the 14,561 cases included in this study, 3,530 (24%) were 15–24 year-old, 4,579 (31%) aged 25–44 years, 4,316 (30%) were 45–64 year-old, and the remaining 2,136 (15%) were 65 years or older.

### Frequency distribution of EPTB

**Figure 1:**
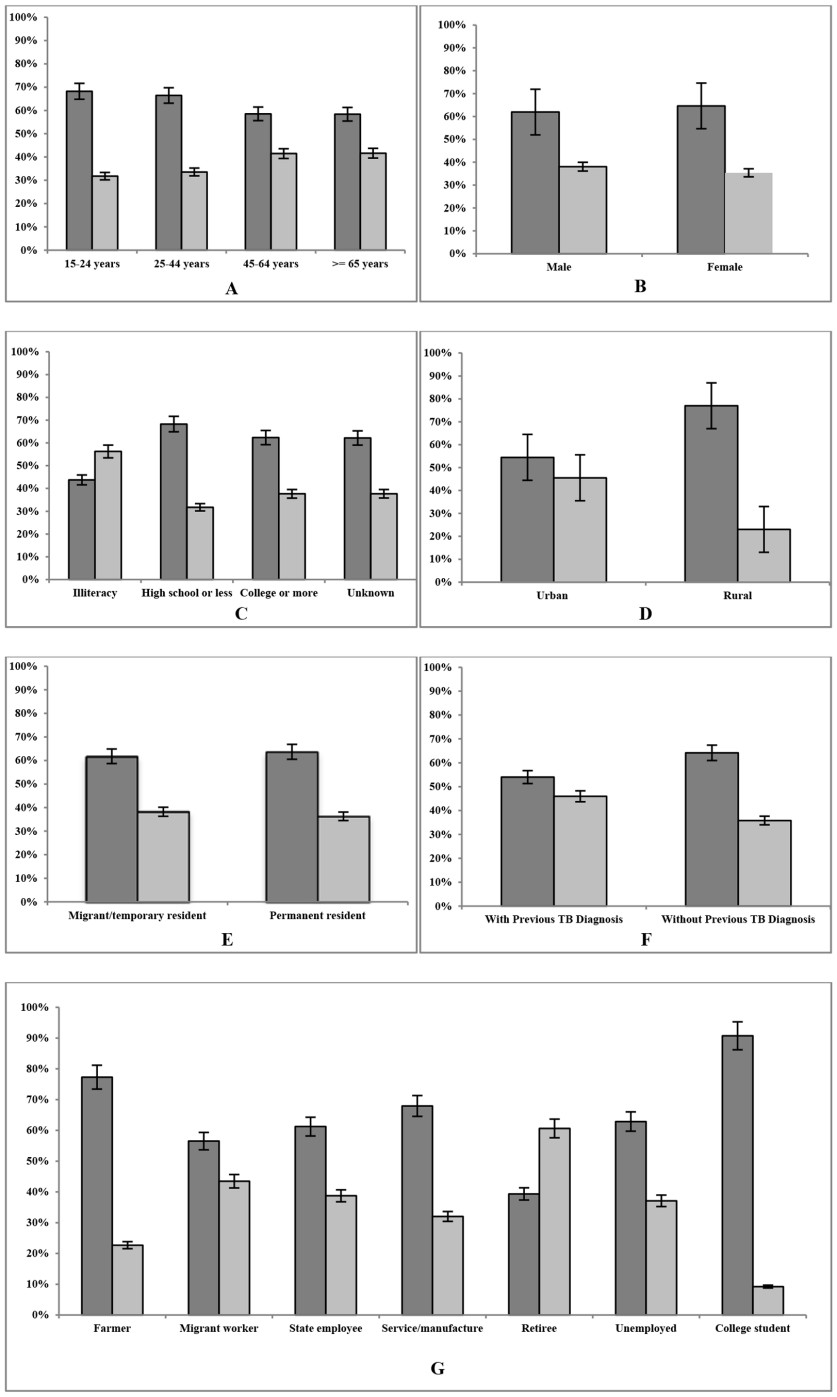
Proportions (y-axis) of extrapulmonary tuberculosis (EPTB) among tuberculosis (TB) patients of different demographic characteristics (x-axis) in Tianjin, China during 2006–2011. A dark gray bar represents pleural TB and a light gray bar represents all the other forms of EPTB. Panel A, by age group; Panel B, by sex; Panel C, by education level; Panel D, by urbanity of residence; Panel E, by stability of residence; Panel F, by history of previous TB diagnosis; Panel G, by occupation. The vertical lines on each bar represent 95% confidence intervals from proportions.

Of the 14,561 TB cases included in the present study, 1,512 (10.3%) had EPTB. Of the 1,512 EPTB cases, 953 (63.0%) had TB pleurisy, while the remaining 559 (37.0%) had TB in anatomic sites outside of thoracic cavity. The proportions of pleural TB and other forms of EPTB varied among patient groups of different age groups (Fig. 1, Panel A), education level (Fig. 1, Panel C), between rural and urban residences (Fig. 1, Panel D), having and not having a history of previous TB diagnosis (Fig. 1, Panel F), and occupations (Fig. 1, Panel G). In contrast, no statistically significant difference in the frequency of pleural TB and other forms of EPTB were observed between males and females (Fig. 1, Panel B) and between permanent and non-permanent residences (Fig. 1, Panel E).

### Comparison of patient characteristics between PTB and EPTB groups

Chi-square analysis of categorical variables of the study patients showed a significant difference in the distribution of age, occupation, and urbanity of residence between PTB and EPTB groups (Table 1). A significantly higher proportion of patients of age 65 year-olds or older were found among EPTB patients than among PTB patients (18.65% vs. 14.21%, p<0.01). In contrast, age group of 25–44 year was found significantly more frequently among PTB patients than among EPTB patients (31.88% vs. 27.67%, p<0.01). For the occupation variable, the most significant differences between PTB and EPTB were related to retirees and farmers. EPTB contained a significantly larger proportion of retirees than PTB (13.71% vs. 8.15%, p<0.01) while PTB was significantly more likely to be farmers than EPTB (39.47% vs. 25.38%, p<0.01). In addition, rural residents were overrepresented in the PTB group while urban residents were overrepresented in the EPTB group. The distribution of sex, education level, stability of residence, and previous TB diagnosis were similar between PTB and EPTB groups (p>0.05, Table 1).

**Table 1:** Distribution of socio-demographic characteristics among pulmonary tuberculosis (PTB) & extrapulmonary tuberculosis (EPTB) patients reported in Tianjin, China during 2006 to 2011.

Characteristics	PTB	EPTB	*P* a
	N (%)	N (%)	
Age (years) (N = 14,561)			<0.01
15–24	3,184 (24.39)	346 (22.96)	
25–44	4,162 (31.88)	417 (27.67)	
45–64	3,853 (29.52)	463 (30.72)	
> = 65	1,855 (14.21)	281 (18.65)	
Sex (N = 14,561)			0.51
Male	8,489 (65.03)	967 (64.17)	
Female	4,565 (34.97)	540 (35.83)	
Education Level (N = 14,561)			0.61
Illiteracy	151 (1.16)	16 (1.06)	
High school or less	2,140 (16.37)	230 (15.26)	
College or more	675 (5.17)	85 (5.64)	
Unknown	10,088 (77.28)	1,176 (78.04)	
Urbanity of Residence (N = 14,561)			<0.01
Rural	5,454 (41.78)	565 (37.49)	
Urban	7,600 (58.22)	942 (62.51)	
Stability of Residence (N = 14,069)			0.75
Migrant/temporary resident	2,502 (19.83)	293 (20.18)	
Permanent resident	10,115 (80.17)	1,159 (79.82)	
Previous TB Diagnosis (N = 14,069)			0.74
With previous TB diagnosis	1,045 (8.28)	124 (8.54)	
Without previous TB diagnosis	11,572 (91.72)	1,328 (91.46)	
Occupation (N = 13,739)			<0.01
Farmer	4,882 (39.47)	348 (25.38)	
Migrant worker	789 (6.38)	92 (6.71)	
State employee	925 (7.48)	129 (9.41)	
Service/manufacture b	1,113 (9.00)	131 (9.56)	
Retiree	1,008 (8.15)	188 (13.71)	
Unemployed	2,402 (19.42)	299 (21.81)	
College student	1,249 (10.10)	184 (13.42)	

a Probability from chi-square analysis.

b Occupations in the food industry, public transportation, public service attendants, and factory workers.

### Risk factors for EPTB

Using EPTB as the case group and PTB as the control group, we assessed the associations between the variables under the study and having EPTB based on crude and multiple logistic regression-derived adjusted odds ratios (OR) and 95% confidence interval (CI) (Table 2).

**Table 2:** Crude and multivariate logistic regression models to determine independent risk factors for having extrapulmonary tuberculosis.

	Crude		Adjusted	
Characteristics	OR	95% CI	OR	95% CI
Age (years)				
15–24	Reference		Reference	
25–44	0.92	0.79–1.07	0.89	0.77–1.04
45–64	1.11	0.96–1.28	1.00	0.86–1.17
> = 65	1.39	1.18–1.65	1.22	1.02–1.46
Sex				
Male	Reference		Reference	
Female	1.04	0.93–1.16	1.04	0.93–1.17
Urbanity of Residence				
Rural	Reference		Reference	
Urban	1.19	1.07–1.34	1.38	1.22–1.55
Occupation				
State employee	Reference		Reference	
Farmer	0.51	0.41–0.63	0.55	0.45–0.69
Migrant worker	0.84	0.63–1.11	0.87	0.65–1.16
Service/manufacture	0.84	0.65–1.09	0.88	0.68–1.14
Retiree	1.34	1.05–1.70	1.37	1.08–1.75
Unemployed	0.89	0.72–1.11	0.95	0.76–1.19
College student	1.06	0.83–1.34	1.12	0.88–1.43

Based on the result of the crude models, age 65 years or older appeared to have a higher risk for having EPTB (OR = 1.39, 95% CI: 1.18, 1.65) than age group of 15–24 year-olds (reference age group), while age groups of 25–44 year-old and 45–64 year-old showed a risk for having EPTB similar to that of the reference age group (Table 2). We chose the age group 15–24 years as the reference group to assess the age association with EPTB because it was the youngest and was considered to be the most healthy group among all the age groups.

For occupation, retirees had statistically significantly higher odds (OR = 1.34, 95% CI: 1.05, 1.70) for having EPTB than the state employees (reference occupation group), while farmers had statistically significantly lower odds (OR = 0.51, 95% CI: 0.41, 0.63) for having EPTB than the state employees. We chose the state employee group as the reference group in assessing the occupation and EPTB association as this group has generally been considered to have the best job security and medical care, compared with other occupation groups analyzed in this study. For urbanity of resistance, urban residents had significantly higher odds (OR = 1.19, 95% CI: 1.07, 1.34) for having EPTB when compared to rural residents.

Further, the multivariate logistic regression models identified three independent predictors for having EPTB diagnosis in Tianjin, China, based on statistically significant adjusted odds ratio (aOR) and 95% CIs. These factors include being age 65 years or older (aOR = 1.22, 95% CI: 1.02, 1.46), being a retiree (aOR = 1.37, 95% CI: 1.08, 1.75,), and residing in urban areas (aOR = 1.38, 95% CI: 1.22, 1.55). In contrast, being a farmer was found to be an independent negative predictor for having EPTB diagnosis. The risk for having EPTB was similar between males and females.

## Discussion

Using TB surveillance data of 14,561 patients, we conducted the first assessment of the relative contribution of PTB and EPTB to the burden of TB in Tianjin, China overall and within subpopulations of different socio-demographic characteristics. We also identified social demographic factors associated with EPTB in the study population.

The major findings of this study were: 1) about one-tenth (10.3%) of the patients had EPTB with majority of the reported EPTB cases being pleural TB; 2) the frequencies of pleural TB and other forms of EPTB differed significantly among different social demographic groups of the patients; and 3) the distribution of age, occupation, and urbanity of residence significantly differed between PTB and EPTB patients were more likely to be 65 years or older, to be retired, and to be living in urban areas were predictors for having EPTB. The proportion of EPTB (10.3%) among all TB cases found in this study was lower than the range reported in some industrialized countries [5]–[9]. Previous studies have reported as high as 21.6%, 38%, and 22% of all TB cases were EPTB in the United States, the Netherlands, and Germany, respectively [9], [20], [21]. Studies conducted in many developed countries have also shown an increase in the proportion of EPTB cases among all TB cases diagnosed in these populations [5]–[10]. However, EPTB is largely undiagnosed in developing countries, due to the difficulties in its diagnosis and the less attention paid to it by National TB Programs because of the perceived less important role of EPTB in TB transmission [3], [12]. Due to the very similar reasons, EPTB was not included in the TB surveillance of China until the mid-1990s. Although EPTB has been included in the TB surveillance of China since the mid-1990s, attention has mainly been paid to pleural TB (Personal communication with Dr. Xiexiu Wang). The National TB Program of China has primarily focused on the reporting of PTB, especially sputum smear positive cases that are most communicable. Most of the TB clinics at county level do not have the ability to diagnose EPTB. Hence, reporting of EPTB was not mandatory in China, except for TB pleurisy, which was reportable and managed as smear negative PTB unless the patient has concurrent sputum smear positive PTB [22]. As a result, it is likely that the observed relatively low proportion of EPTB in our study population is, at least, partly due to the under-diagnosis of EPTB, especially EPTB outside of the thoracic cavity, in Tianjin, China. Previous studies have reported the effect of sex, race/ethnicity, age, and HIV infection on the risk of an individual for having EPTB [13]–[15], [23]–[27]. However, because a limited attention has been given to EPTB by TB control programs in developing countries, most previous studies on EPTB risk factors were conducted in developed countries. There is little information about EPTB epidemiology in developing countries, including China. This study extends the knowledgebase about EPTB in developing countries by identifying several host social-demographic characteristics as the predictors for having EPTB diagnosis.

In this study, being age 65 years or older was found to be an independent predictor for having EPTB. The observed age association with EPTB is consistent with findings from previous studies conducted in other populations [23], [28]. Similar to findings of the study conducted in Denmark by Zhang et al. (2011), when compared to age group of 15–24 year-olds, the odds for having EPTB was significantly lower for age groups 25–44 year-olds and 45–64 year-olds [23], [29], [30]. However, it is interesting to note that unlike in previous studies where females were found to have higher odds for having EPTB than males [9], [23], in the present study, we did not find a female sex EPTB association. The reasons for our failing to find the previously reported female sex and EPTB association remain to be investigated. In addition, the retirees were found to have higher odds for having EPTB when compared to state employees. This is the first observation of such an association.

The other intriguing findings of this study are that farmers had lower odds for having EPTB compared to state employees and that coincidently, the rural residents had lower odds for having EPTB than the urban residents. Given that the majority of farmers resided in the rural areas of Tianjin where the medical capacity for EPTB diagnosis is more limited than that in urban areas of Tianjin, the observed negative associations between EPTB and farmers and rural resident are most likely due to the potential under-diagnosis of EPTB among these two subpopulations.

As mentioned above, one of the major limitations of the study is the inadequacy in EPTB diagnosis and reporting has limited our ability to describe the trends of EPTB as well as the frequency of different forms of EPTB, one of the most important questions for EPTB epidemiology study. Furthermore, because the TB surveillance data that we used for this study only recorded one disease type for each patient, patients who had concurrent PTB and EPTB might have been recorded as having PTB alone, given that PTB is often the prerequisite of EPTB and is more likely to be recognized by physician than EPTB during the diagnosis of TB. This potential misclassification of cases with concurrent pulmonary and extrapulmonary involvement as PTB cases might have resulted in an underassessment of the potential associations of certain patient characteristics with EPTB. The associations identified in this study would have been stronger if there had not been such misclassification in this study. Furthermore, studies have shown that individuals have a higher risk for developing EPTB other than pleural TB than immune competent individuals, as their immune system is not strong enough to contain TB within the thoracic cavity [31]. EPTB outside of thoracic cavity and pleural TB may represent different process of pathogenesis. Therefore, the risk factors for having EPTB identified in our study may not represent all the risk factors for other forms of EPTB rather than pleural TB, given the sample bias potentially introduced by the under detection of EPTB outside of thoracic cavity in our study.

The current study also had several other limitations that might have resulted in an inadequate adjustment of potential confounders and mediators in the identification of the risk factors for EPTB in this study. First, information about study subjects' HIV/AIDS status, a well-documented risk factor for EPTB [29], was not available for the time period that the current study covered, although the screening of HIV infection among TB patients has been conducted more routinely in more recent years. Nevertheless, we believe that the confounding effect of HIV status on the data analysis in our study was minor given the low prevalence of HIV infection among TB patients reported for China (0.9%) based a recent meta-analysis involving 29 studies conducted in China [32]. In addition, co-morbidity information was also very limited. Therefore, the immune status of the study patient that may impact on the risk of the patients for having EPTB was not adjusted for in the data analyses. The study is also limited in that information on *Mycobacterium tuberculosis* factors that were previously reported to be associated with EPTB was as not available for the present study [33], [34].

However, despite these limitations, as the first study of EPTB in Tianjin, China using a large dataset, this study generated useful new knowledge about EPTB epidemiology in China. Since EPTB cases have been under-diagnosed in developing countries, including China, the findings of this study are useful to the development of improved EPTB detection in China by more targeted screening of EPTB among high-risk populations and strengthening EPTB diagnostic capacity of TB clinics in rural areas of the country. The present study also highlighted several areas in China's TB surveillance that should be improved to maximize the usefulness of the TB surveillance data for TB epidemiological studies and disease monitoring.
